# Deciphering the Duality of Clock and Growth Metabolism in a Cell Autonomous System Using NMR Profiling of the Secretome

**DOI:** 10.3390/metabo6030023

**Published:** 2016-07-26

**Authors:** Arjun Sengupta, Saikumari Y. Krishnaiah, Seth Rhoades, Jacqueline Growe, Barry Slaff, Anand Venkataraman, Anthony O. Olarerin-George, Chi Van Dang, John B. Hogenesch, Aalim M. Weljie

**Affiliations:** 1 Department of Systems Pharmacology and Translational Therapeutics, Perelman School of Medicine, University of Pennsylvania, Philadelphia, PA 19104, USA; arjunsen@upenn.edu (A.S.); yksaikumari@gmail.com (S.Y.K.); srhoades@mail.med.upenn.edu (S.R.); jgrowe0923@gmail.com (J.G.); barry.slaff@gmail.com (B.S.); anandven22@gmail.com (A.V.); olarerin@gmail.com (A.O.O.-G.); hogenesch@gmail.com (J.B.H.); 2 Institute of Translational Medicine and Therapeutics, Perelman School of Medicine, University of Pennsylvania, Philadelphia, PA 19104, USA; 3 Abramson Family Cancer Research Institute, Perelman Schol of Medicine, University of Pennsylvania, Philadelphia, PA 19104, USA; dangvchi@exchange.upenn.edu; 4 Department of Molecular and Cellular Physiology, University of Cincinnati, Cincinnati, OH 45267, USA

**Keywords:** NMR spectroscopy, metabolomics, circadian oscillations, U2 OS, detrending

## Abstract

Oscillations in circadian metabolism are crucial to the well being of organism. Our understanding of metabolic rhythms has been greatly enhanced by recent advances in high-throughput systems biology experimental techniques and data analysis. In an in vitro setting, metabolite rhythms can be measured by time-dependent sampling over an experimental period spanning one or more days at sufficent resolution to elucidate rhythms. We hypothesized that cellular metabolic effects over such a time course would be influenced by both oscillatory and circadian-independent cell metabolic effects. Here we use nuclear magnetic resonance (NMR) spectroscopy-based metabolic profiling of mammalian cell culture media of synchronized U2 OS cells containing an intact transcriptional clock. The experiment was conducted over 48 h, typical for circadian biology studies, and samples collected at 2 h resolution to unravel such non-oscillatory effects. Our data suggest specific metabolic activities exist that change continuously over time in this settting and we demonstrate that the non-oscillatory effects are generally monotonic and possible to model with multivariate regression. Deconvolution of such non-circadian persistent changes are of paramount importance to consider while studying circadian metabolic oscillations.

## 1. Introduction

The physiology and behavior of most complex organisms is regulated in a circadian manner. Chronic disruption of this rhythm can lead to a cascade of maladaptive effects including cognitive and psychiatric dysfunction, metabolic disorders and cancer [1,2,3,4,5,6,7]. Transcriptionally, cell autonomous circadian rhythms are generated via core clock genes. Briefly, BMAL1 and CLOCK are transcriptional activators that regulate the transcriptional repressors Cryptochrome (CRY1 and CRY2) and Period (PER1 and PER2). PER and CRY proteins, on the other hand, inhibit the BMAL1/CLOCK function post translationally, and this activation–inhibition loop in turn generates the oscillatory rhythms in gene expression [8]. In addition to transcriptional oscillation, cellular metabolism also shows inherent oscillatory rhythm.

The human osteosarcoma cell line, U2 OS, is used as a convenient model system as the core clock genes are well-characterized and constitute a cell-autonomous molecular oscillator [9]. Study of biological rhythms in metabolites is a recent development in this regard [10] and are indicative of potentially important phenomena. For example, recent results from our group showed that ectopic MYC oncogene expression significantly affects oscillation in glucose in metabolism and glutaminolysis [11]. While studies of such rhythms are increasingly important, the metabolic context of general metabolite changes and metabolic status over the sampling time required for high time-resolution circadian rhythm studies has not been described. In other words, the media of cell culture growth may contain elements of both the circadian metabolic phenomena in addition to general metabolite changes due to cell growth and change in cell numbers.

Under ideal conditions, circadian measurements of cell autonomous systems would require the metabolic health of the cells under study to remain uniform throughout the course of the experiment, such that only oscillations due to clock-dependent changes are observed. In practice, we hypothesize that in the common setup for cell culture measurements of circadian characteristics, two temporal metabolic factors are in play. First, the innate circadian rhythm of the cellular metabolism (Circadian process) and second, a linear continuous change of the cellular metabolism over the two days of collection dictated by the properties of the secretome, i.e. nutrient availablity and waste metabolite build-up (Metabolic process). We investigated these effects by profiling the metabolites of the cell culture media using nuclear magnetic resonance (NMR) spectroscopy. NMR, although relatively insensitive, offers a robust and quantitative approach to study global metabolism [12]. Metabolomics using NMR was enhanced using the targeted profiling approach [13], which takes advantage of spectral fitting using a library of compound spectra and an internal standard to obtain absolute concentrations of metabolites in the sample. Thus, NMR-based metabolomics offers a quantitative window to study the changes in temporal cellular metabolism in the context of a cell autonomous oscillator. Our results indicate continuous change in metabolite composition of the media over time resulting from cellular metabolic changes. These results suggest that analysis of oscillatory metabolic rhythms must be corrected for linear growth/secretion effects.

## 2. Results

### 2.1. Temporal Change of the Global Secretome Composition in a Cell-Autonomous Model System

**Figure 1:**
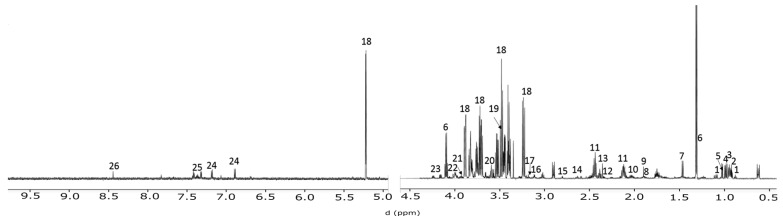
^1^H-NMR spectral assignment of DMEM media. A total 26 metabolites were quantified using targeted profiling. 1—3-Methyl-2-oxovalerate, 2—2-Oxoisocaproate, 3—Leucine, 4—Isoleucine, 5—Valine, 6—Lactate, 7—Alanine, 8—Lysine, 9—Acetate, 10—Pyroglutamate, 11—Glutamine, 12—Glutamate, 13—Pyruvate, 14—Methionine, 15—Methylguanidine, 16—cis-Aconitate, 17—Choline, 18—Glucose, 19—Glycine, 20—myo-Inositol, 21—Serine, 22—Fructose, 23—Threonine, 24—Tyrosine, 25—Phenylalanine, 26—Formate.

**Figure 2:**
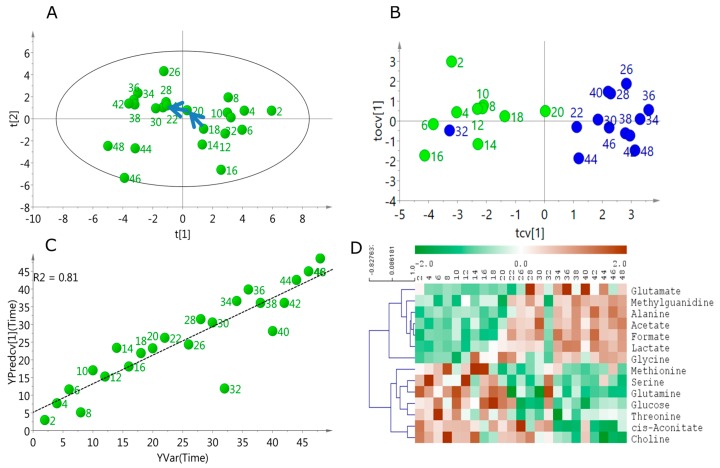
Multivariate modeling of DMEM (Dulbecco’s Modified Eagle Medium) media metabolic profiles: (**A**) PCA scores plot demonstrate a temporal shift in the cellular metabolic profile from 18 h to 22 h as note by the arrows; (**B**) (Orthogonal Partial Least Square-Discriminant Analysis) OPLS-DA scores plot showing distinct clustering between the pre- and post-shift time points; (**C**) OPLS modeling using time as the regressor variable led to a robust predictive model as suggested from the strong linearity between actual time points and those predicted from the model; and (**D**) heat map and hierarchical cluster of the metabolites significantly related to time points from supervised multivariate modeling (Both OPLS-DA and OPLS).

Synchronized U2 OS cells were cultured and the media sampled every two hours over 48 h, followed by NMR spectroscopic analysis. Twenty-six metabolites from the media were profiled (Figure 1), and interrogated by multivariate analysis. Principal component analysis (PCA) indicated significant time

dependent variation in the metabolic profile of the media. Scores from the 1st PC were evidently associated with sample collection timepoint (Supporting Information S1). For example, the variation along PC1 described the continuous change in the media profile, while PC2 showed an oscillatory pattern with an approximate period of 28 h (using eye estimate, no statistical analysis was performed). The scores plot from the PCA of the cultured media samples over 48 h showed two distinct groups divided approximately by circadian days. Interestingly, a shift from Day 1 to Day 2 was evident around 18–22 h of sampling time (Figure 2A).

Further orthogonal partial least squares discriminant analysis (OPLS-DA) modeling was performed using the pre-shift (2 h–18 h) and post-shift (20 h–48 h) samples. The model was robust (CV-ANOVA *p* = 0.0004, Q^2^ (cum) = 0.66) and the scores plot indicated a distinct clustering of the pre- and post-shift samples (Figure 2B). Interestingly, the pre- and post-shift samples were well separated with the exception of the sample collected at 32 h. Metabolites that were significantly different between the two classes were selected based on the multivariate variable influence on projection (VIP, >1.0) and are listed in Table 1. We observed increase in glutamate, methylguanidine, alanine, acetate, formate, lactate and glycine. Concomitant decrease was observed in methionine, serine, glutamine, glucose, threonine, cis-Aconitate, and choline. Interesting trends include reverse temporal trends in glutamate–glutamine and glucose–lactate. Generally, temporal increase in metabolic excretory products such as lactate, methylguanidine, acetate and formate was observed while several metabolic precursors including amino acids and glucose decreased over time.

The temporal analysis was done using two different multivariate models: OPLS-DA (for pre and post time variation) and OPLS (for correlation of metabolites with time). In both cases, metabolites with VIP (Variable Importance on Projection) > 1.0 were judged significant. In the left panel, negative loadings indicate increased level before the shift time, while, in the right panel, negative loading indicates linear decrease in level with time.

### 2.2. Linear Temporal Changes in the Secretome

**Figure 3:**
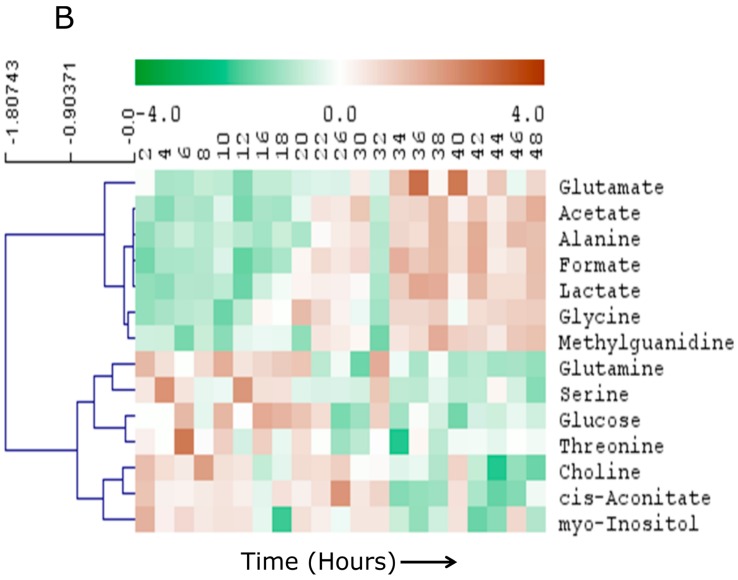
Heat map demonstrating temporal trends of the 14 metabolites in media that were significantly altered during the time course. Metabolites are clustered using HCA with distance calculated by Pearson correlation. Each box of the heat map represents time in hours.

We reasoned that there would be linear time-dependent changes in media composition as a function of both nutrient depletion and metabolite excretion. In order to test this hypothesis, metabolites that were linearly changing over the time course of sampling was obtained using multivariate OPLS modeling with time of collection as the *Y*-variable. A robust (CV-ANOVA *p* = 2.66 × 10^-6^, Q^2^ (cum) = 0.81) and strongly predictive OPLS model was generated (Figure 2C). The metabolites (VIP > 1.0) uniformly changing with time are listed in Table 1. Univariate analysis showed 14 significantly (*p* < 0.05, FDR < 0.1) altered metabolites post 22 h timepoints. These included glutamate, acetate, alanine, formate, lactate, glycine, methylguanidine (all elevated post 22 h), glutamine, serine, glucose, threonine, choline, cis-aconitate, and myo-inositol (all decreased post 22 h). Relative levels of these metabolites are shown in Figure 3, *p*-values and FDRs of individual metabolites are listed in Table 2.

Metabolites significantly changing between pre- and post-shift time points in the U2 OS cells and media were identified using permutation based unpaired *t*-test.

### 2.3. Specific Temporal Metabolic Changes in the Secretome

**Figure 4:**
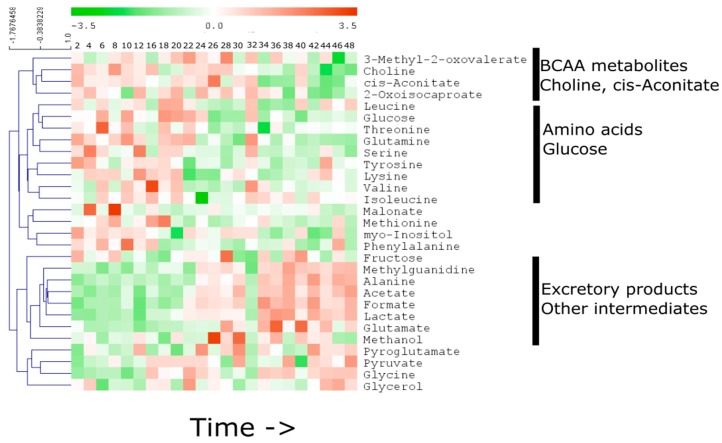
Temporal profile of all media metabolites over the complete 48 h time course. The metabolites are clustered as in Figure 3. Each box of the heat map represents time in hours.

Five metabolites from the media samples showed a particularly strong linear correlation with time. These metabolites are acetate, alanine, choline, lactate and methylguanidine. Interestingly, with the exception of choline, the other metabolites are end products of metabolic pathways. These four metabolites were increased linearly (*r* for lactate = 0.88, acetate = 0.87, alanine = 0.88, formate = 0.90 and methylguanidine = 0.74, for all of them *p* < 0.01) over time while choline showed linear depletion in the media (*r* = −0.77, *p* < 0.01). To further understand the changes in the metabolites in the media, the total media metabolite profile was subjected to hierarchical cluster analysis (Figure 4). The metabolites were segregated into five clusters.

Major clusters include: (1) Metabolites of branched chain amino acids, choline and cis-aconitate; (2) amino acids and glucose; (3) malonate, methionine, myo-inositol and phenylalanine; (4) end products of cellullar metabolism; and (5) pyroglutamate, pyruvate, glycine and glycerol. The metabolites in these five clusters showed grossly similar temporal behavior (Supplementary Information S2). For example, level of branched chain amino acid (BCAA) metabolites remained broadly the same longitudinally, while choline was steadily decreased in the media. Several amino acids and glucose level in the media were depleted during 18–24 h time while the metabolic end products were steadily increased in the media.

## 3. Discussion

Physiological circadian rhythms are an important component of organismal well-being, and disruption of the internal body clock can lead to detrimental and pathophysiological effects [14]. This is followed directly from recent literature related to biological rhythms in different systems [15,16,17]. Many of these studies investigate the problem using systems biology approaches [18,19], for example by studying large-scale transcript or protein profiles. For cellular systems under study, however, the temporal cellular metabolism will have two distinct components including the metabolic oscillation and cell growth/excretion factors, which are primarily linear in nature. Decoupling of these two components is crucial for time resolved analysis such as circadian metabolic studies. In this work, we attempted to investigate the linear changes of cellular metabolome by profiling the cell culture media secretome in a setting that is used for investigating circadian metabolic/gene oscillations.

While intra-cellular metabolism can be complex and difficult to study, the culture media of the cell line under study could provide significant information about the physiological activity within the cell. This approach has been utilized for numerous cell lines including red blood cells [20] and hepatocytes [21]. Some of the findings may have potential therapeutic applications. For example, Drago et al. reviewed the potential of manipulation of stem cell secretome as an important pathway of therapeutic strategy for brain repair [22]. Therapeutic potential of secreome, specifically in context of metabolomics, were also demonstrated in non-invasive embryo assessment during in vitro fertilization (IVF) [23]. At a more fundamental level, bacterial culture media was recently used to demonstrate the effect of metal toxicity in *Pseudomonas pseudoalcaligenes* [24]. However, little is known about the metabolic effect of long time course experiment in the media found typically in a setting of circadian metabolic studies. In this work, we show that metabolic profiling of media could be used to gain insights of cellular metabolism in a system relevant for studying circadian rhythm in mammalian cell lines.

Our data suggest that the metabolic composition of media in this U2 OS system undergoes a smooth transition during 18–22 h of experiment (Figure 2). We note that this transition occurs within the context of a cell-confluent system. This is important as sub-confluent systems have been shown to demonstrate a combination of growth and circadian changes in core clock gene expression [25]. Wavelets based methods have been suggested for recovering information from a growing cell system that is otherwise not observed [26]. Our data suggested that a group of metabolites including precursor molecules such as amino acids and glucose were decreased after 22 h while several cellular end products were increased (Figure 3). Detailed temporal tracking of the metabolites indicated that the media level of several precursor amino acids and glucose underwent depletion specifically between 18 and 22 h (Supplementary Information S2). This effect suggests that the cellular consumption of metabolic precursor molecules is not continuous. Instead, the consumption is probably pulsatile and synchronized for different metabolic pathways. However, the catabolic end products of cellular metabolism (lactate, acetate, alanine, formate and methylguanidine) build up continuously in the media (Figure 4), suggesting the catabolic activity of cellular metabolism is not pulsed. This observation further raises the possibility that cellular metabolic oscillation is controlled, at least partially, by the temporal availability of precursor molecules. Indeed, we have observed specific cellular metabolic oscillations in U2 OS cells [10], which could be the result of pulsed cellular intake of media metabolites. In addition, such obvious exchange of metabolites across plasma membrane suggests that a more complex flux based models may be needed to elucidate the metabolic clock mechanism in U2 OS cells. Further analysis would require more sensitive analysis however as the inherent insensitivity of NMR technique does not allow for testing this hypothesis for relatively less abundant metabolites.

Overall, our data suggest that a considerable amount of cellular chronobiology can be unraveled by profiling the metabolites of the cell culture media. Specifically, the cellular growth and catabolism related activities can be efficiently followed using NMR spectroscopy. As the NMR data are highly quantitative and robust, it is an ideal platform from which to study temporal changes. The present study, as well as our previous work [11], demonstrates that NMR spectroscopy can be utilized to that end. The nature of cellular circadian rhythm could be further unraveled by profiling the cellular metabolites. In addition, as noted, we did not observe any circadian rhythms in the media metabolites, which could be an obvious limitation of the NMR platform. More sensitive techniques such as LC-MS are needed for further investigation of oscillating media metabolites. Nevertheless, we have shown that linear effects of cellular metabolism are highly prominent in a typical setting to investigate circadian effects in such in vitro systems. Therefore, these effects should be accounted for while investigating oscillatory metabolic activities in the cell.

## 4. Materials and Methods

### 4.1. U2OS Cell Collection

Cells were prepared as previously described [27]. Briefly, U2 OS cells were seeded at 5 million cells per 10 cm dishes in DMEM medium containing 10% fetal bovine serum (FBS) without antibiotics. Cells were allowed to adhere to the surface for 24 h and synchronized with 0.1 μM dexamethasone. The collection began 24 h post-synchronization. During the collection, media was collected and snap frozen every two hours for 48 h [11].

### 4.2. Metabolite Extraction from Media

Fifty microliters of media was thawed on ice and metabolites were extracted using a modified Bligh–Dyer method [28,29]. Briefly, a methanol:chloroform (2:1) mixture (300 μL) was added to the cell pellets, then vortexed and sonicated for 15 min. Chloroform and water (100 μL each) were then added and samples were vortexed. Organic and aqueous layers were separated by centrifugation at 13,300 rpm, for 7 min at 4 °C. The aqueous layer was dried and reconstituted in 200 μL phosphate buffer (pH 7.0) in 10% D_2_O (Cambridge Isotope Laboratories, Andover, MA, USA) containing 4,4-dimethyl-4-silapentane-1-sulfonic acid (DSS) as internal standard (Cambridge Isotope Limited). The samples were taken in 3 mm NMR tube (Bruker Biospin, Billerica, MA, USA) for spectral acquisition.

### 4.3. NMR Spctroscopy

All spectral acquisitions were performed in Bruker Avance III HD NMR spectrometer, equipped with a triple resonance inverse (TXI) 3 mm probe (Bruker Biospin). Bruker Samplejet was used for sample handling to ensure high throughput nature. The pulseprogram took the shape of first transient of a 2 dimensional NOESY and generally of the form RD-90-t-90-tm-90-ACQ [12]. Where RD = relaxation delay, t = small time delay between pulses, tm = mixing time and ACQ = acquisition. Water signal was saturated using continuous irradiation during RD and tm. The spectra were acquired using 76 K data points and 14 ppm spectral width. Sixty-four scans were performed and 1 s interscan (relaxation) delay and 0.1 s mixing time was allowed. The FIDs were zero filled to 128 K; 0.1 Hz of linear broadening was applied followed by Fourier transformation, baseline and phase correction using an automated program provided by Bruker Biospin.

### 4.4. Spectral Profiling and Data Analysis

Raw FID from ^1^H-NMR was processed and profiled using Chenomx NMR suite 8.0. ^1^H-NMR data were evaluated using a targeted profiling strategy [13] that allows quantification of metabolite data in the sample.

Pre-processed data were exported to SIMCA-P 14 (Umetrics AB, Umea, Sweden) for further multivariate statistical analysis. Univariate scaling was appliedto put eqal weightage to all variables. Initially, unsupervised Principal Component Analysis (PCA) was carried out to look for trends in metabolites. Further, supervised clustering was performed using Orthogonal Partial Least Square-Discriminant Analysis (OPLS-DA). In addition, regression of metabolome with time was assessed using supervised OPLS modeling. Supervised models were internally cross-validated with a 7-fold cross validation. These models were considered significant for models with cross-validated ANOVA *p* < 0.05. Variables were selected based on variable importance of projection (VIP), and VIP > 1 was considered significant. Due to non-availability of one time point sample (14th time point), the missing data were replaced by averaging preceding (12th) and succeeding (16th) time points.

Univariate analysis was carried out using MeV 4.9, with permutation-based t tests. Variables were considered significant for *p* < 0.05 and false discovery rate (FDR) < 0.1 correcting for multiple testing.

## Figures and Tables

**Table 1:** Media metabolites with significant temporal trends from 0–48 h.

OPLS-DA Model (Pre- and Post-Time Shift)			OPLS Model (Time-Dependent)		
Metabolite	VIP	P (loadings)	Metabolite	VIP	P (loadings)
Glutamine	1.42	−0.28	Glutamine	1.37	−0.25
Glucose	1.28	−0.25			
Serine	1.20	−0.24	Serine	1.08	−0.19
Threonine	1.06	−0.21	Cis-Aconitate	1.10	−0.22
Methionine	1.06	−0.20			
Choline	1.05	−0.17	Choline	1.36	−0.27
Glycine	1.06	0.15	Glycine	1.25	0.25
Glutamate	1.06	0.22	Glutamate	1.17	0.22
Methylguanidine	1.17	0.22	Methylguanidine	1.35	0.27
Alanine	1.33	0.24	Alanine	1.55	0.30
Acetate	1.37	0.26	Acetate	1.55	0.31
Lactate	1.41	0.26	Formate	1.60	0.31
Formate	1.53	0.30	Lactate	1.61	0.32

**Table 2:** Significantly altered metabolites identified by univariate analysis.

Media					
Increased Pre-Shift	*P*	FDR	Increased Post-Shift	*p*	FDR
Glutamine	0	0	Glutamate	0.002	0.01
Serine	0.01	0.03	Acetate	0	0
Glucose	0.004	0.02	Alanine	0	0
Threonine	0.005	0.02	Formate	0	0
Choline	0.004	0.02	Lactate	0.002	0.01
cis-Aconitate	0.04	0.08	Glycine	0.02	0.04
myo-Inositol	0.05	0.1	Methylguanidine	0.005	0.02

## Supplementary Materials

The Supplementary Information is available online at www.mdpi.com/ 2218-1989/6/3/23/s1; Supplementary Information S1: Changes of PC-scores over time (0–48 h). The PCA modeling was done on the profiled data from the cell culture media from U2OS cells. First three PCs are shown. Supplementary Information S2: Temporal changes of all cell culture media metabolites. Profiled concentration (μM, *Y*-axis) of all 26 metabolites are plotted against time (*X*-axis).
