# Supplementary figures and images for: Deciphering the Duality of Clock and Growth Metabolism in a Cell Autonomous System Using NMR Profiling of the Secretome
Source: Metabolites. 2016 Jul 26;6(3):23. doi: 10.3390/metabo6030023 (PMC5041122; doi:10.3390/metabo6030023)

**PC1**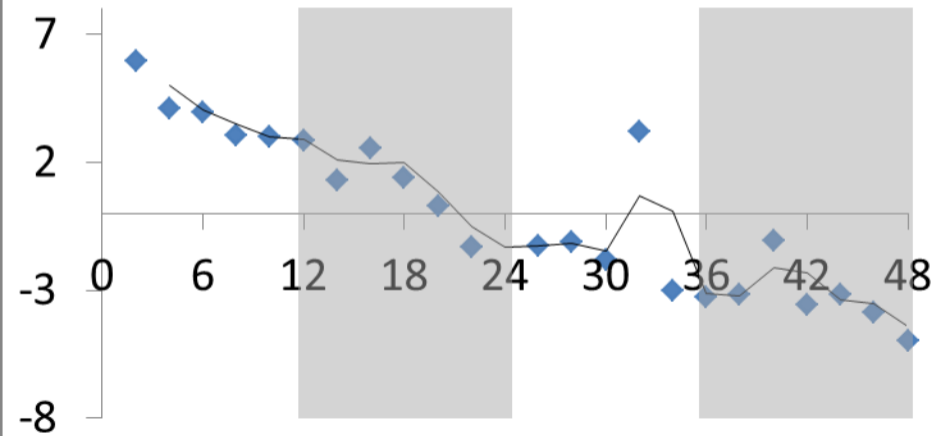**PC2**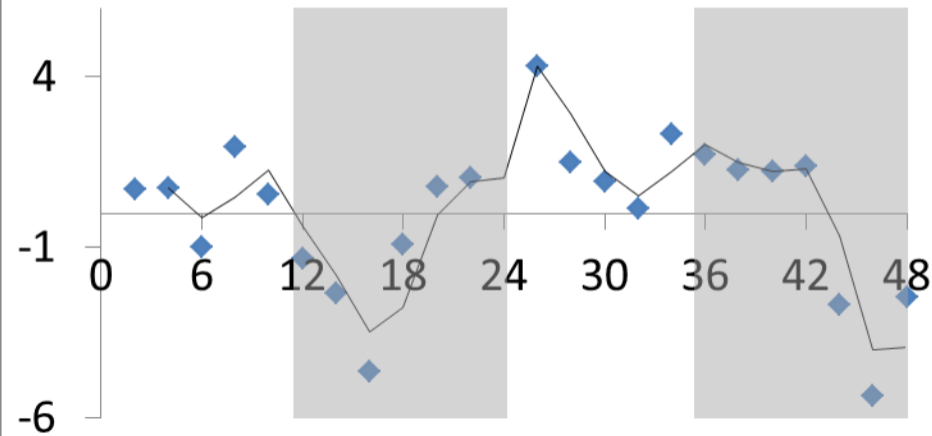**PC3**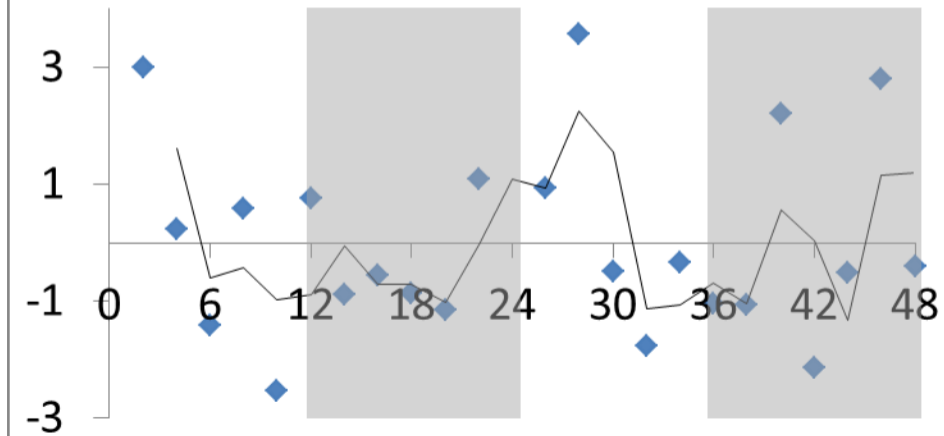

Supplement: Supplementary file 1 [file metabolites-06-00023-s001.zip › Supp-Info-S1.pdf]

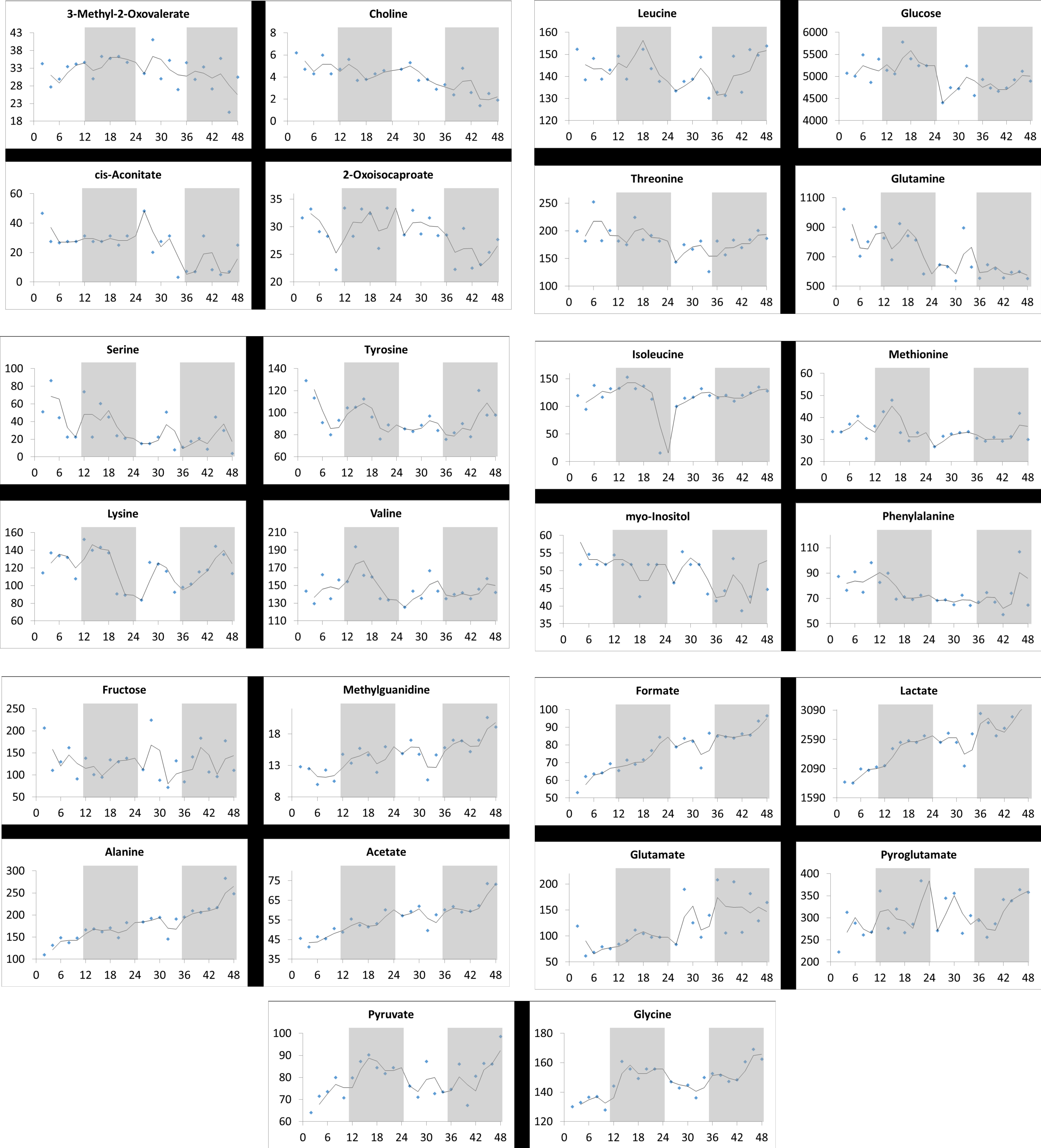

Supplement: Supplementary file 1 [file metabolites-06-00023-s001.zip › Supp-Info-S2.pdf]
